# Supplementary material for: Mesenchymal stromal cells immunosuppress osteoarthritis synovial fluid modulated monocytes via IL-6 and CCL2
Source: Cell Death Dis. 2026 Aug 14;17(1):887. doi: 10.1038/s41419-026-09194-y (PMC13669379; doi:10.1038/s41419-026-09194-y)
Supplement: Supplementary file 1 [file 41419_2026_9194_MOESM1_ESM.pdf]

Supplementary figures 1-8

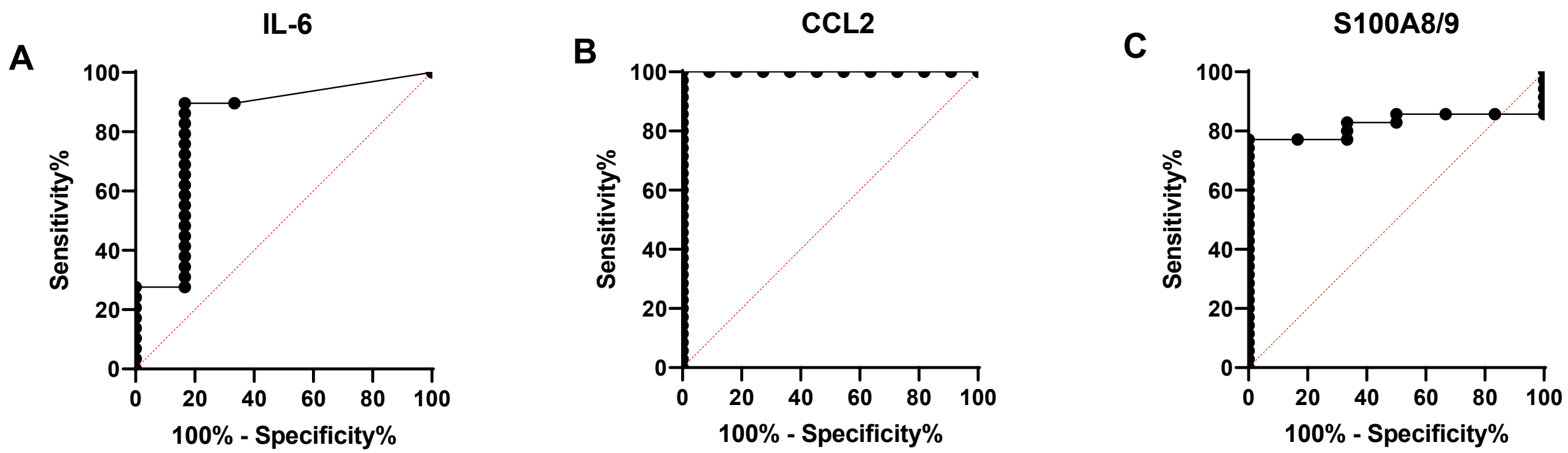

Figure S1. IL-6, CCL2, and S100A8/9 are specifically increased in late OA SF, as indicated by the increased area under the receiver operating characteristic (ROC) curves

(A-C) ROC curves indicated the specificity of IL-6, chemokine (C-C motif) ligand 2 (CCL2), and S100A8/9 measurements in late-OA synovial fluid (SF) compared to control, non-diseased SF samples; diagonal represents a random classifier.

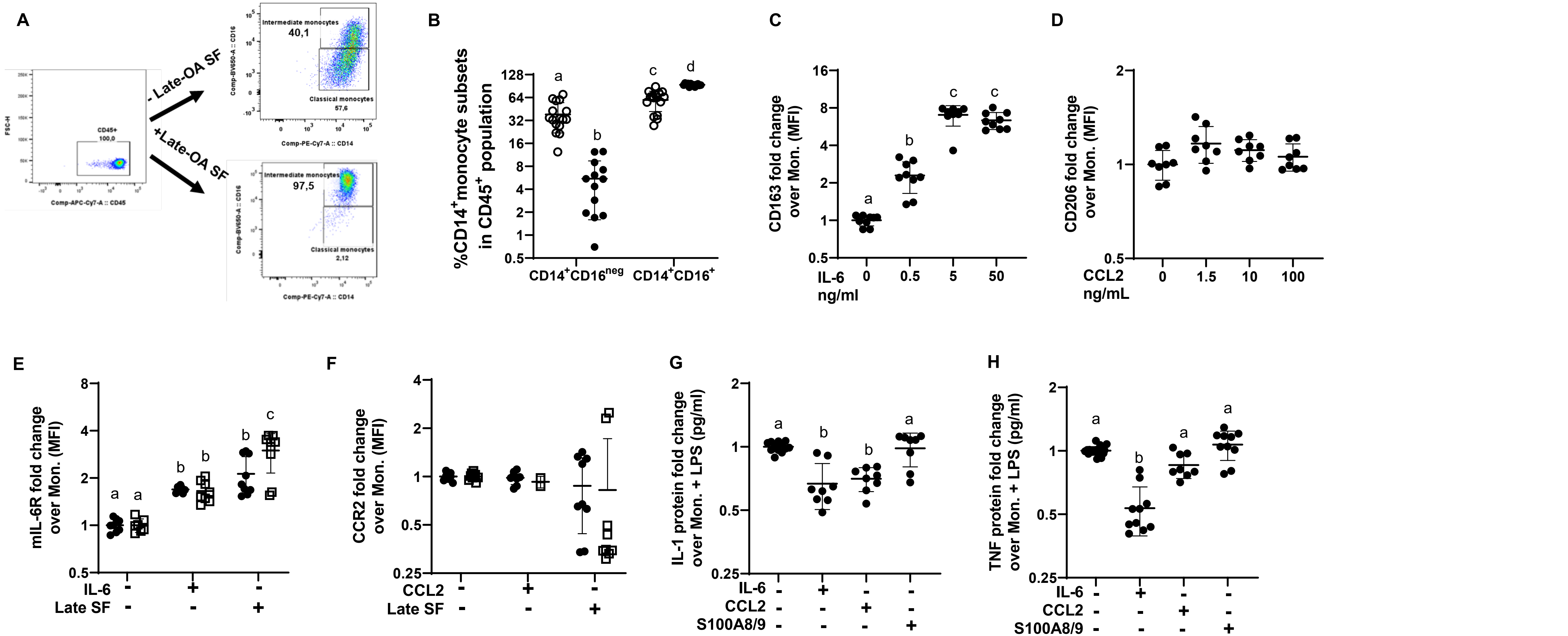

Figure S2. Late-OA SF, IL-6, CCL2 and S100A8/9 effects on peripheral CD14⁺ monocytes

(A, B) Frequency of classical (CD14⁺CD16^neg^) and intermediate (CD14⁺CD16⁺) peripheral healthy CD14⁺ monocyte subsets with (●) and without (○) OA synovial fluid (SF). Dose-response of healthy, peripheral CD14⁺ monocytes on **(C)** CD163 MFI with different IL-6 concentrations or on **(D)** CD206 MFI with different CCL2 concentrations. (**E, F**) membrane IL-6R (mIL-6R) and CCR2 expression on healthy (●) or KOA (□) CD14⁺ monocytes at indicated treatments. **(G, H)** TNF and IL-1 soluble factor production by CD14⁺ monocytes at indicated treatments, normalized to control LPS stimulation. N=3 biological replicates; n=3 technical replicates. Letters indicate significant differences based on an ordinary one-way ANOVA followed by Tukey's multiple comparisons test.

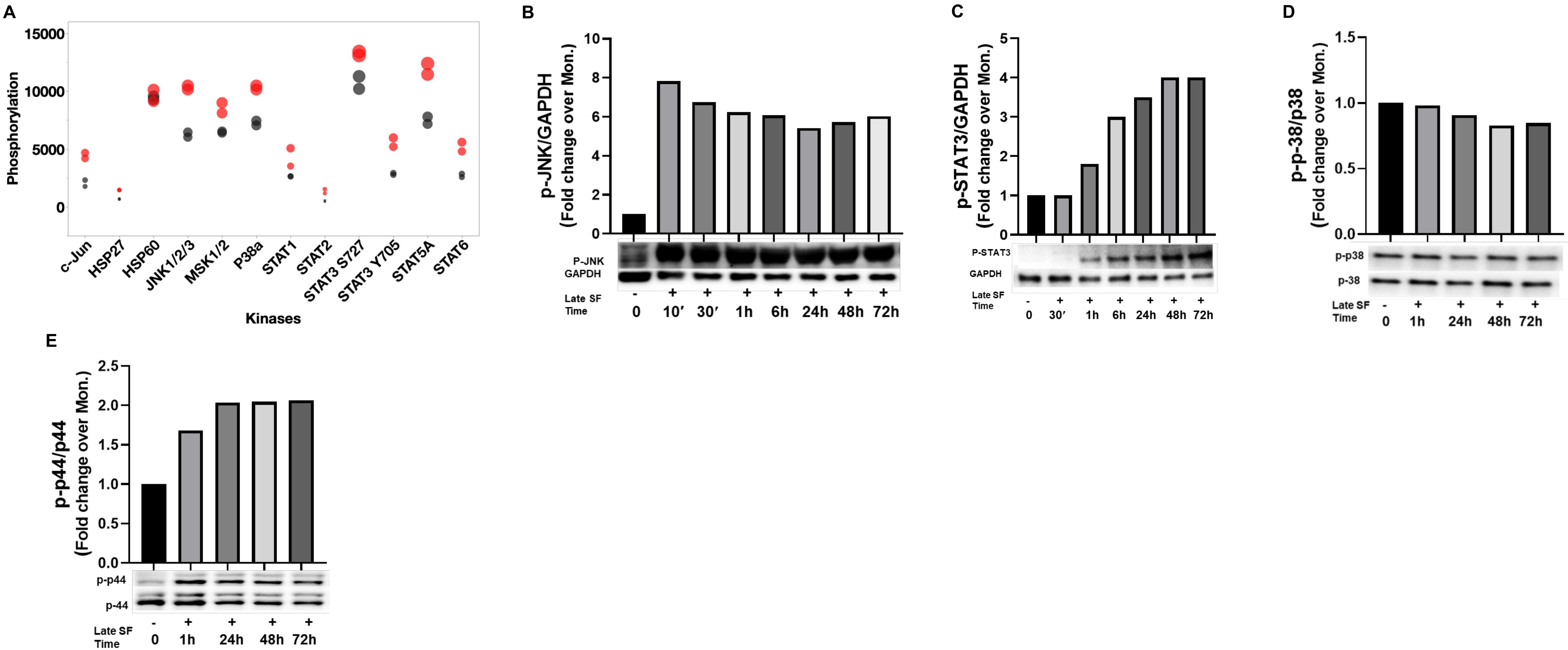

Figure S3. Late-OA SF activates different signaling pathways in CD14⁺ monocytes

(A) Bubble plot of the phosphorylation profiles of kinases in CD14⁺ monocytes treated with (red) and without late-OA SF (gray) with bubble size representing signal intensity corresponding to the level of phosphorylated kinase protein (N=1 biological replicates; n=2 technical replicates; small (<5000 pixels); medium (5000-10000 pixels); large (10000- 15000 pixels)). **(B-E)** Representative Western blots and bar graphs for phosphorylated (p)-JNK, p-STAT3 levels (normalized to GAPDH), and p-protein (p)38 levels (normalized to p38), p-p44 levels (normalized to p44) at the indicated times (N=1 donor, n=3 technical replicates). HSP27; Heat shock protein 27, HSP60; Heat shock protein 60, MSK 1/2; Mitogen and stress-activated kinases 1/2.

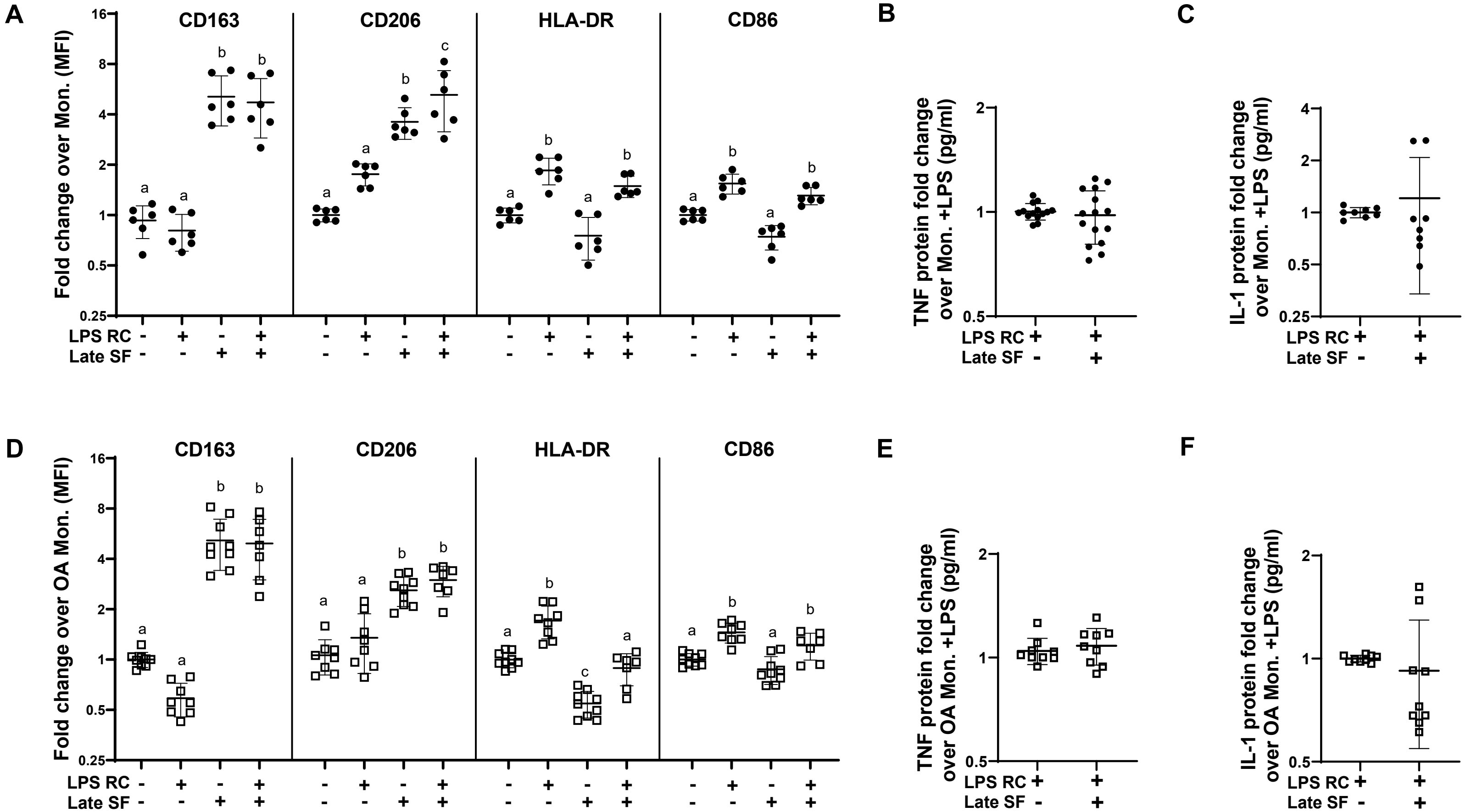

Figure S4: CD14⁺ monocytes rechallenged with LPS in late-OA SF

(A, D) CD163, CD206, HLA-DR, and CD86 MFI in healthy (●) or KOA (□) CD14⁺ monocytes exposed to pooled late OA SF after LPS re-challenge (RC)**. (B, E)** TNF, and **(C, F)** IL-1 soluble factors production from healthy (●) or KOA (□) peripheral CD14⁺ monocytes after LPS RC in late-OA SF. N=3 biological replicates; n=3 technical replicates. Letters indicate significant differences based on an ordinary one-way ANOVA followed by Tukey's multiple comparisons test.

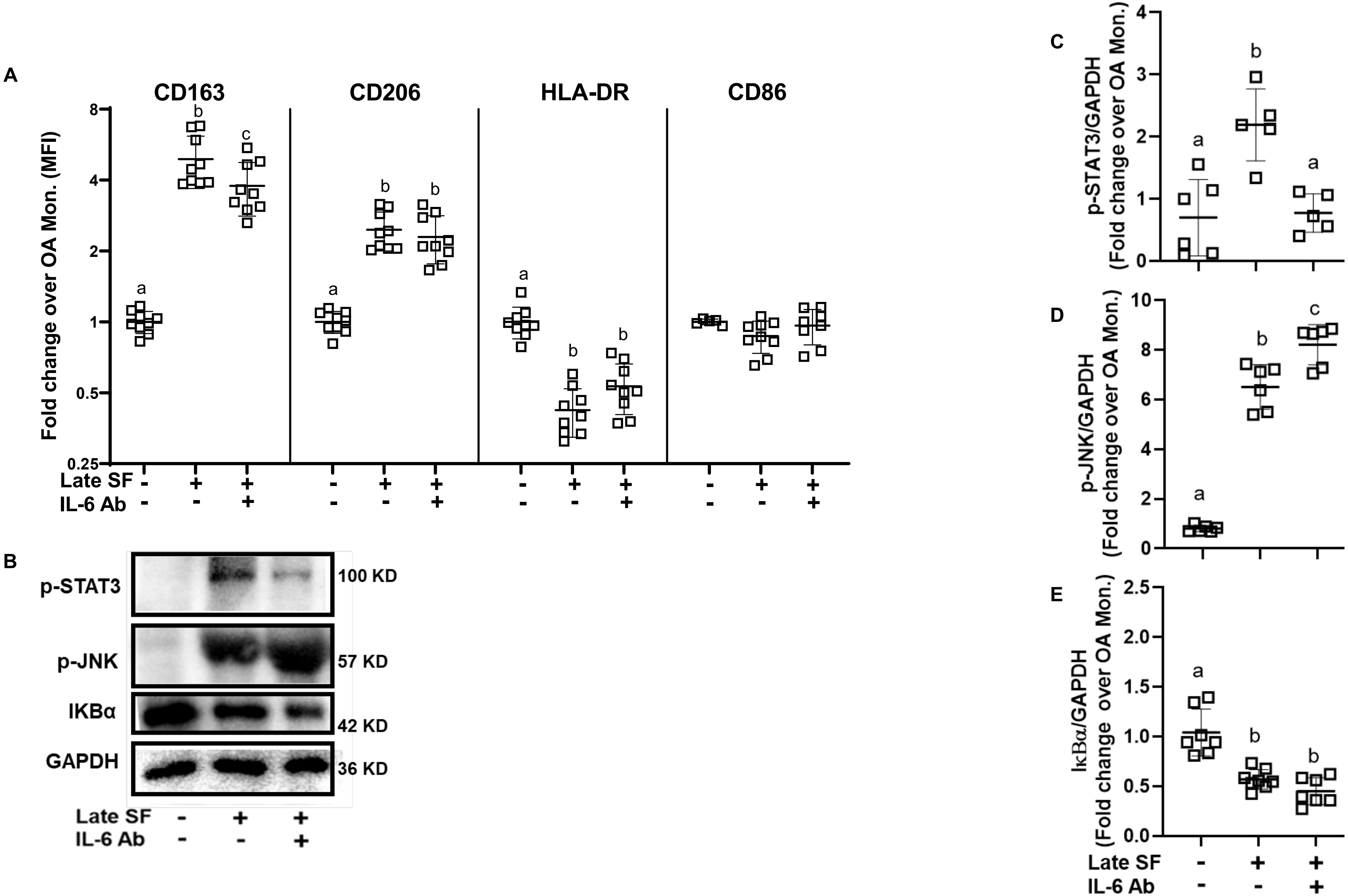

Figure S5. Late-OA synovial fluid effects on KOA patient-sourced peripheral CD14⁺ monocytes

(A) MFI of CD163, CD206, HLA-DR, and CD86 on KOA peripheral CD14⁺ monocytes at indicated treatments. **(B)** Representative Western blot. **(C-E)** Bar graphs of p-STAT3, p-JNK, and IκBα levels (normalized to GAPDH). N=3 biological replicates; n=3 technical replicates. Letters indicate significant differences based on an ordinary one-way ANOVA followed by Tukey's multiple comparisons test.

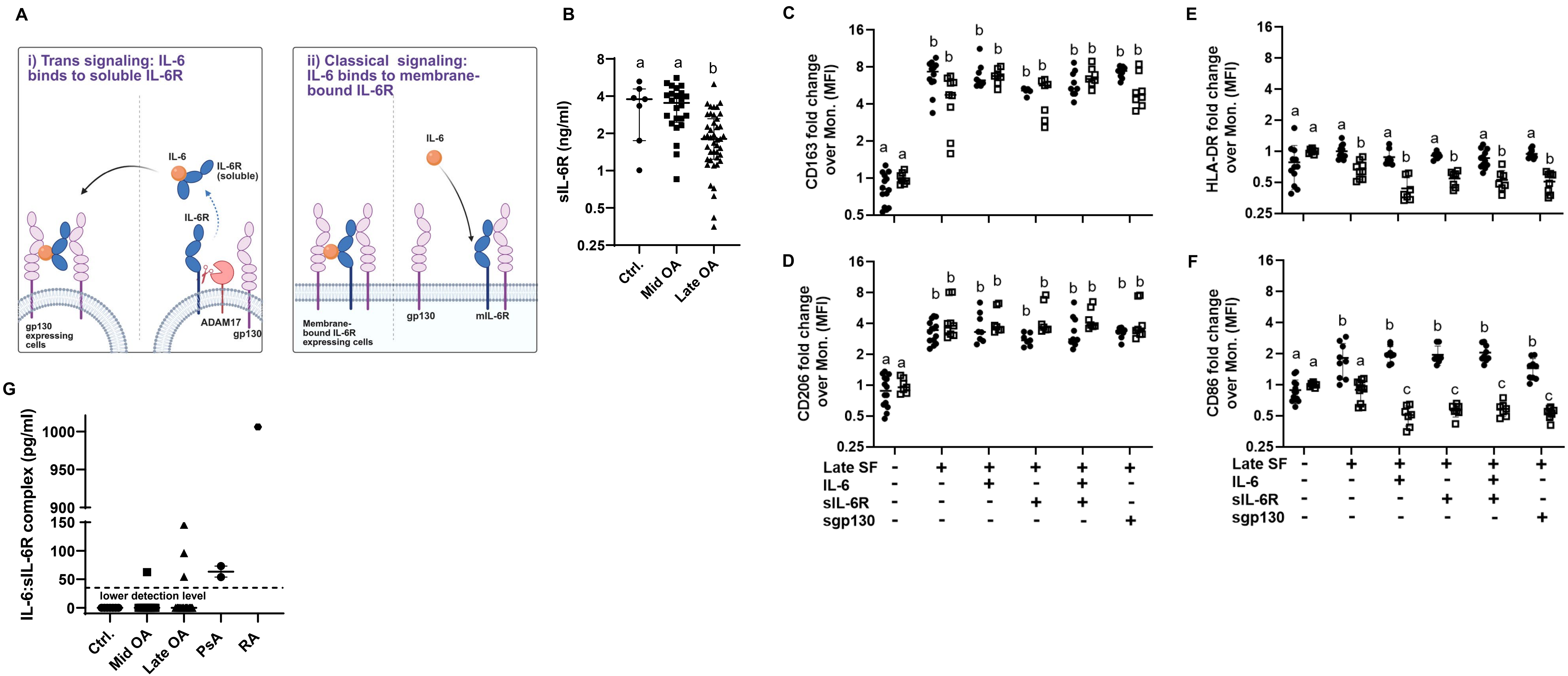

Figure S6. Physiological levels of IL-6 in KOA SF signal through classical, not trans-signaling

(A) IL-6 signaling in monocytes/MΦs: i) trans-signaling through glycoprotein (gp)130 and soluble IL-6R (sIL-6R shed by a disintegrin and metalloproteinase 17 (ADAM 17)); ii) classical signaling through membrane-bound (m)IL-6R and gp130. **(B)** Median and interquartile range of sIL-6R concentration in SF from ctrl. (non-diseased, N=7); mid-OA (N=24); and late-OA (N=43). **(C-F)** MFI expression of CD163, CD206, HLA-DR, and CD86 at indicated treatments in healthy (●) and KOA (□) CD14^{+} monocytes; N=3 biological replicates; n = 3 technical replicates. **(G)** IL-6:sIL-6R complex levels in ctrl. (non-diseased; N=7); mid-OA (N=24); late-OA (N=72), psoriatic arthritis (PsA) SF (N=2) and rheumatoid arthritis (RA) SF (N=1). Letters indicate significant differences based on an ordinary one-way ANOVA followed by Tukey's multiple comparisons test.

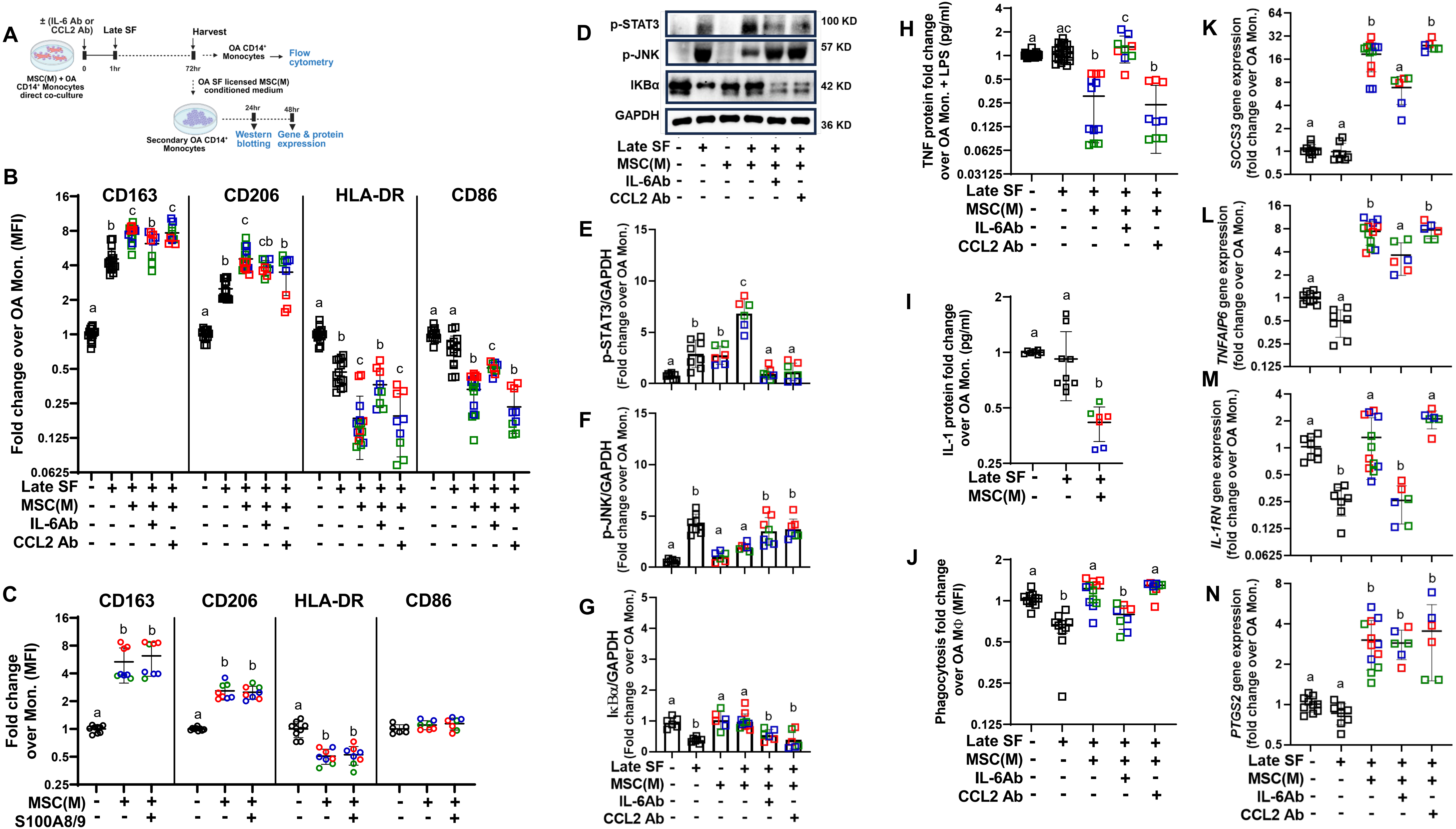
A
± (IL-6 Ab or CCL2 Ab)
Late SF
Harvest
MSC(M) + OA CD14⁺ Monocytes direct co-culture
0
1hr
72hr
OA CD14⁺ Monocytes
Flow cytometry
OA SF licensed MSC(M) conditioned medium
24hr
48hr
Secondary OA CD14⁺ Monocytes
Western blotting
Gene & protein expression
B
Fold change over OA Mon. (MFI)
CD163
CD206
HLA-DR
CD86
Late SF
MSC(M)
IL-6Ab
CCL2 Ab
C
Fold change over Mon. (MFI)
CD163
CD206
HLA-DR
CD86
MSC(M)
S100A8/9
D
p-STAT3
p-JNK
IKBα
GAPDH
100 KD
57 KD
42 KD
36 KD
Late SF
MSC(M)
IL-6Ab
CCL2 Ab
E
p-STAT3/GAPDH (Fold change over OA Mon.)
F
p-JNK/GAPDH (Fold change over OA Mon.)
G
IκBα/GAPDH (Fold change over OA Mon.)
Late SF
MSC(M)
IL-6Ab
CCL2 Ab
H
TNF protein fold change over OA Mon. + LPS (pg/ml)
Late SF
MSC(M)
IL-6Ab
CCL2 Ab
I
IL-1 protein fold change over OA Mon. (pg/ml)
Late SF
MSC(M)
J
Phagocytosis fold change over OA MΦ (MFI)
Late SF
MSC(M)
IL-6Ab
CCL2 Ab
K
SOCS3 gene expression (fold change over OA Mon.)
L
TNFAIP6 gene expression (fold change over OA Mon.)
M
IL-1RN gene expression (fold change over OA Mon.)
N
PTGS2 gene expression (fold change over OA Mon.)
Late SF
MSC(M)
IL-6Ab
CCL2 Ab

Figure S7. Direct co-culture of MSC(M) with KOA CD14⁺ monocytes in late-OA SF

(A) Experimental schematic of KOA $CD14^+$ monocytes directly co-cultured with MSC(M); late-OA SF or IL-6/CCL2 neutralizing antibodies. **(B)** MFI of CD163, CD206, HLA-DR, and CD86, with or without late-OA SF. **(C)** CD163, CD206, HLA-DR, and CD86 MFI of healthy $CD14^+$ monocytes co-cultured with MSC(M) in the presence of S100A8/9. **(D)** Representative Western blot. **(E-G)** p-STAT3, p-JNK, and IκBα levels (normalized to GAPDH). **(H, I)** Normalized TNF and IL-1 soluble factors production at indicated treatments. **(J)** Phagocytosis, at indicated treatments. **(K-N)** Expression of *SOCS3*, *TNFAIP6*, *IL-1RN, PTGS2 genes* . MSC(M) donors are represented by different colors (blue, green, red). N=3 biological replicates; n=3 technical replicates. Letters indicate significant differences based on an ordinary one-way ANOVA followed by Tukey's multiple comparisons test.

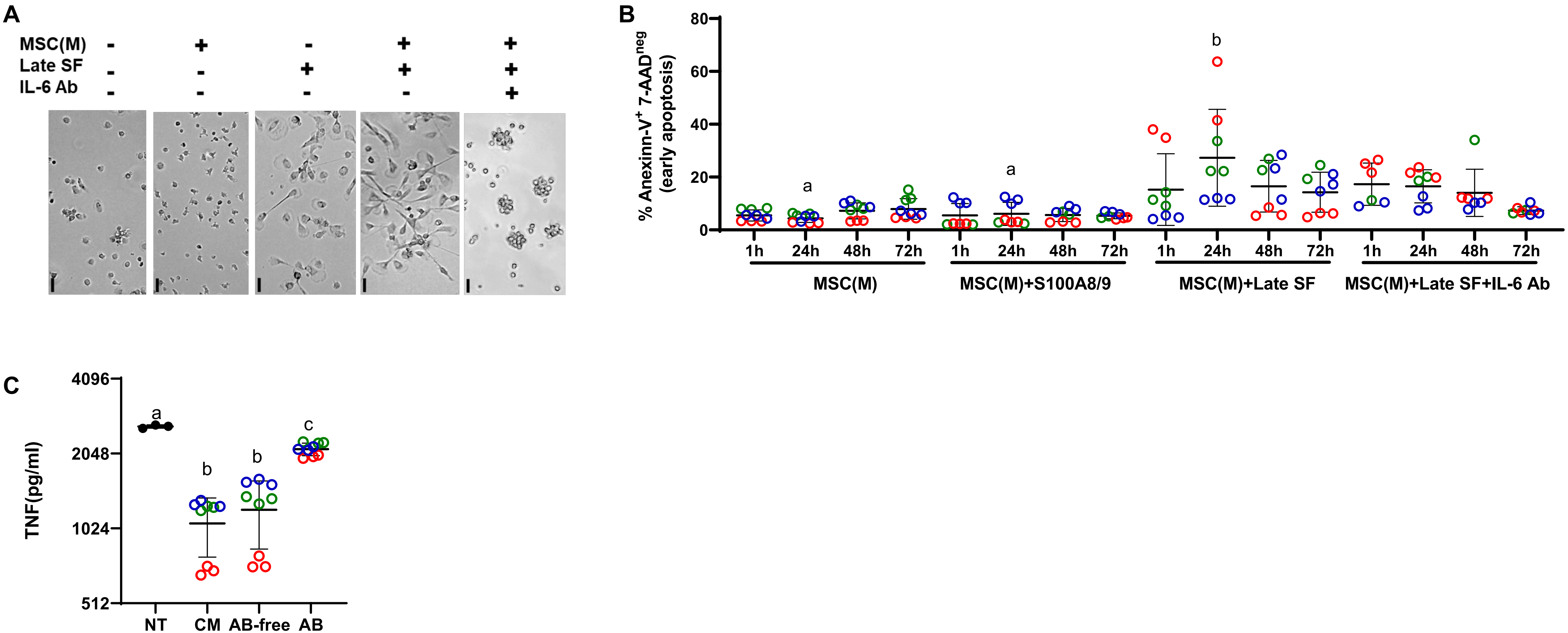

Figure S8. Effects of MSC(M) soluble factors on secondary CD14⁺ monocytes

(A) Representative CD14⁺ monocyte images at indicated treatments; 20x objective; scale bar, 10 μm. **(B)** Apoptosis frequency in MSC(M) treated with S100A8/9, late-OA SF, and late-OA LSF with IL-6-neutralizing antibody (Ab) at different time points. **(C)** TNF soluble factor production in response to different fractions of the licensed MSC(M) conditioned medium (CM), NT (untreated control), AB (apoptotic bodies), and AB-free CM. MSC(M) donors are represented by different colors (blue, green, red). N=3 biological replicates; n=3 technical replicates. Letters indicate significant differences based on an ordinary one-way ANOVA followed by Tukey's multiple comparisons test.

Supplementary Tables

Tables S1-S9

Table S1. Demographics of KOA patients SF donors MSC(M) donors

Variable	Mid OA patients (N = 35)	Late OA patients (N = 104)	MSC(M) donors (N=3)
**BMI, Median (IQR)**	29.51 (25.7-32.3) kg/m^2	29.34 (2.32 - 33.88) kg/m^2	25.2, 28, 29 kg/m^2
**Age, Median (IQR)**	57 (52 - 65) years	65.5 (61 - 72) years	32, 50, 58 years
**Male N, %**	18 (51%)	44 (42%)	3 (100%)
**KL**	II, Early III	Late III, IV	Healthy

Table S2. IL-6, CCL2, and S100A8/9 measurements in SF

IL-6 pg/mL			CCL2 pg/mL			S100 A8/9 ng/mL		
control N=6	Mid OA N=21	Late OA N=29	control N=11	Mid OA N=27	Late OA N=35	control N=6	Mid OA N=30	Late OA N=35
23.12	0	0	30.05	652.2	354.8	240.77	101.50	1762.60
0	33.99	540.09	26.37	597.6	934.6	179.11	195.60	1564.99
0	34.19	611.74	120.84	606.9	372.4	184.45	122.64	207.29
439.64	73.6	690.3	103.07	312.9	723.7	187.34	190.30	940.49
0	75.435	151.11	62.6	347.4	701.4	256.81	74.41	272.42
0	275.24	88.34	70.7	1112.4	1392.4	205.22	110.27	387.76
	0	67.14	28.11	246	548.9		160.07	1368.60
	119.70	93.95	23.77	464.5	332.1		291.71	1178.33
	0	232.06	119.59	335.8	412.1		127.49	1111.85
	67.37	306.06	79.59	255.6	208.3		193.56	597.42
	37.29	481.65	74	516.3	447.8		861.93	942.93
	293.55	108.66		559.4	1248.1		1142.32	116.61
	730.93	150.71		305.8	279		181.64	272.69
	0	327.90		813.4	287.4		160.77	435.72
	688.88	233.10		181.2	846.1		111.24	1035.43
	0	0		58.9	268.7		223.6	261.86
	449.21	338.95		112.2	1353.4		112.79	841.19

	445.37	758.07		1007.1	451.4		26.62	160.90
	40.2	76.28		162.9	812.6		360.68	666.70
	0	60.74		101.6	159.3		69.401	427.80
		51.015		216.4	299.9		134.71	348.71
		31.09		329.9	243.9		628.67	114.81
		895.52		597.3	427.8		250.07	453.05
		0		268.1	983.7		1003.08	193.06
		584.85		290.9	155.1		771.81	105.03
		752.935		190.8	242		544.68	205.94
		297.65		225.2	235.2		167.42	1377.94
		119.785			278.8		384.44	325.29
		175.65			475.1		857.72	1756.22
					715.8		142.71	1948.25
					435.7			372.39
					334.6			1080.34
					937.5			859.42
					122.9			493.54
					338.9			105.7

Table S3. CD14$^+$ monocytes from healthy or KOA patients in response to late OA SF

Measure	Healthy CD14$^+$ Monocytes + Late-OA SF rel. to untreated controls	OA CD14$^+$ Monocytes +Late-OA SF rel. to untreated controls
**CD163 MFI**	↑	↑
**CD206 MFI**	↑	↑
**HLADR MFI**	↔	↓
**CD86 MFI**	↔	↔
**mIL-6R MFI**	↑	↑
**CCR2 MFI**	↔	↔
**pSTAT3/GAPDH**	↑	↑

**pJNK/GAPDH**	↑	↑
**IKB/GAPDH**	↓	↓
SOCS3	↔	↔
TNFAIP6	↔	↔
IL1RN	↔	↓
PTGS2	↔	↔
**TNF secretion**	↔	↔
**IL1 secretion**	↔	↔
**Phagocytosis**	↓	↓

Table S4: Analysis of CD14⁺ monocytes with tested stimulators

	**Rel. to untreated CD14⁺ Monocytes**					**Rel. to Late-OA SF treated CD14⁺ Monocytes**		
	**Late-OA SF**	**IL-6**	**CCL2**	**S100A8/9**	**IL-6 + CCL2 + S100A8/9**	**IL-6 Ab**	**CCL2 Ab**	**TAK242**
**CD206**	++	+	=	=	=	=	-	=
**CD163**	+++	+	=	=	=	-	=	=
**HLA-DR**	=	=	=	=	=	=	=	=
**CD86**	=	=	=	=	=	=	=	=
**p-STAT3**	+	+	=	=	=	--	-	=
**p-JNK**	++	=	=	=	=	+	=	=
**IκBα**	-	=	=	-	=	-	=	+
**TNF**	=	NA	NA	NA	-	+	=	-
**IL-1**	=	NA	NA	NA	=	+	=	=
**Phagocytosis**	-	NA	NA	NA	-	=	=	=

Table S5: Analysis of MSC(M) effects on CD14⁺ monocytes

	**Rel. to Late-OA SF**	**Rel. to MSC + Late-OA SF treated cells**	
	**Effect of MSC + LSF**	**Effect of IL-6 Ab**	**Effect of CCL2 Ab**
**CD206**	+++	=	-
**CD163**	++++	-	-
**HLA-DR**	-	+	=

**CD86**	-	=	=
**p-STAT3**	++	--	--
**p-JNK**	-	++	++
**IκBα**	+	-	-
**IL-1**	-	NA	NA
**TNF**	-	++	=
**Phagocytosis**	+	-	=
IL1RN	+	-	=
TNFAIP6	+	-	=
PTGS2	+	=	=
SOCS3	+	--	-

Table S6. Antibodies for immunophenotyping CD14$^+$ peripheral monocytes and SF monocytes/MΦs

**Antibodies**	**Source**	**Dilution**	**Catalogue number (Cat#)**
APC-Cy7 anti-human CD45	BioLegend	1:100	Clone #HI30; Cat# 304014
PE-Cy7 anti-human CD14	BioLegend	1:100	Clone #M5E2; Cat# 982510
BV650 anti-human CD16	BioLegend	1:50	Clone #3G8; Cat# 302042
FITC anti-human CD163	BioLegend	1:50	Clone #GHI/61; Cat# 333618
BV421 anti-human CD206	BioLegend	1:50	Clone #15-2; Cat# 321126
PerCP-Cy5.5 anti-human HLA-DR	BioLegend	1:100	Clone #L243; Cat# 980414
PerCP-Cy5.5 anti-human mIL6Ra	BioLegend	1:50	Clone #UV4; Cat# 352812
PE anti-human TNF antibody	BioLegend	1:50	Clone #MAb11; Cat# 502909
Pacific Blue anti-human IL-1	BioLegend	1:50	Clone #H1b-98; Cat# 511710
CCR2	BioLegend	1:50	Clone #KO36C2; Cat# 357210

Table S7. ELISA kits to detect soluble factors in SF and conditioned medium

**Soluble factors**	**Source**	**Dilution Factor**	**Catalogue number**
IL-6	Biolegend	SF,1:5	430504
CCL2	Biolegend	SF,1:25	438804
S100A8/9	R&D Systems	SF,1:250	DY8226-05
IL6-sIL6R	R&D Systems	SF, not diluted	DY8139-05
sIL-6R	My BioSource	SF,1:2	MBS266072
IL-1	Biolegend	CM, not diluted	437004
TNFα	R&D Systems	CM,1:2	DY210-05

Table S8. Antibodies for immunoblotting

Antibodies	Source	Catalogue number
Phospho-STAT3-Y705 Rabbit mAb	ABclonal	AP0705
Phospho-JNK1/2-T183/Y185 Rabbit mAb	ABclonal	AP0473
Phospho-p38 MAP-T180/Y182 Rabbit mAb	ABclonal	AP0526
P38 MAPK Rabbit mAb	Cell Signaling	9212
p-p44/42 (T202/Y204) (E10) mouse Ab	Cell Signaling	9106
P44/42 MAPK Erk1/2 (137F5) Rabbit mAb	Cell Signaling	4695
IKB (L35A5) mouse Ab	Cell Signaling	4814
GAPDH (14C10) Rabbit mAb	Cell Signaling	2118

Table S9. Primer sequences

Genes name	Forward sequence	Reverse sequence
GAPDH	GGAGCGAGATCCCTCCAAAAT	GGCTGTTGTCATACTTCTCATGG
B2M	CTCCGTGGCCTTAGCTGTG	TTTGGAGTACGCTGGATAGCCT
β-Actin	CTCACCATGGATGATGATATCGC	AGGAATCCTTCTGACCCATGC
PTGS2	ATAAGCGAGGGCCAGCTTTC	CGCAGTTTACGCTGTCTAGC
SOCS3	TTCGGGACCAGCCCCC	AACTTGCTGTGGGTGACCAT
TNFAIP6	AGCACGGTCTGGCAAATACA	ATCCATCCAGCAGCACAGAC
IL-1RN	AGC ATG AGG CTC AAT GGG TA	AAA TCC AGC AAG ATG CAA GC
