# Supplementary material for: Mesenchymal stromal cells immunosuppress osteoarthritis synovial fluid modulated monocytes via IL-6 and CCL2
Source: Cell Death Dis. 2026 Aug 14;17(1):887. doi: 10.1038/s41419-026-09194-y (PMC13669379; doi:10.1038/s41419-026-09194-y)

Uncorrupted WB images

Figure 2

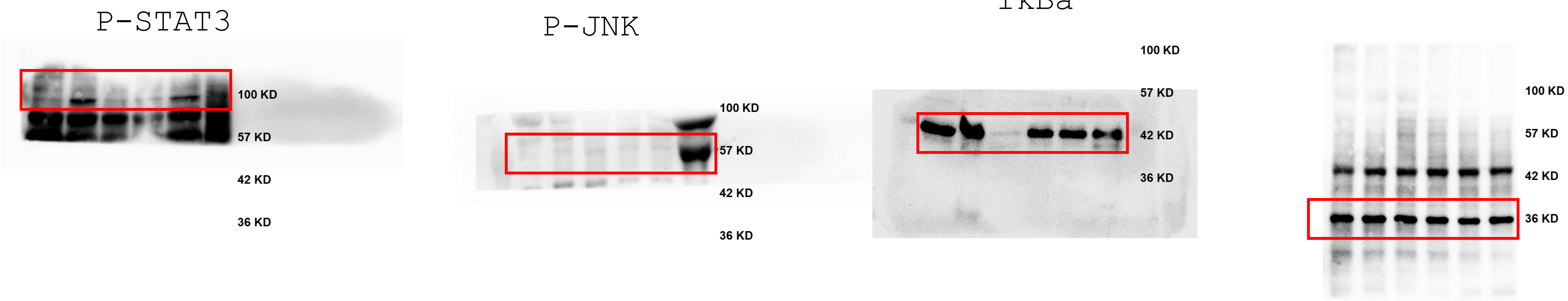

Uncorrupted WB images

Figure 3

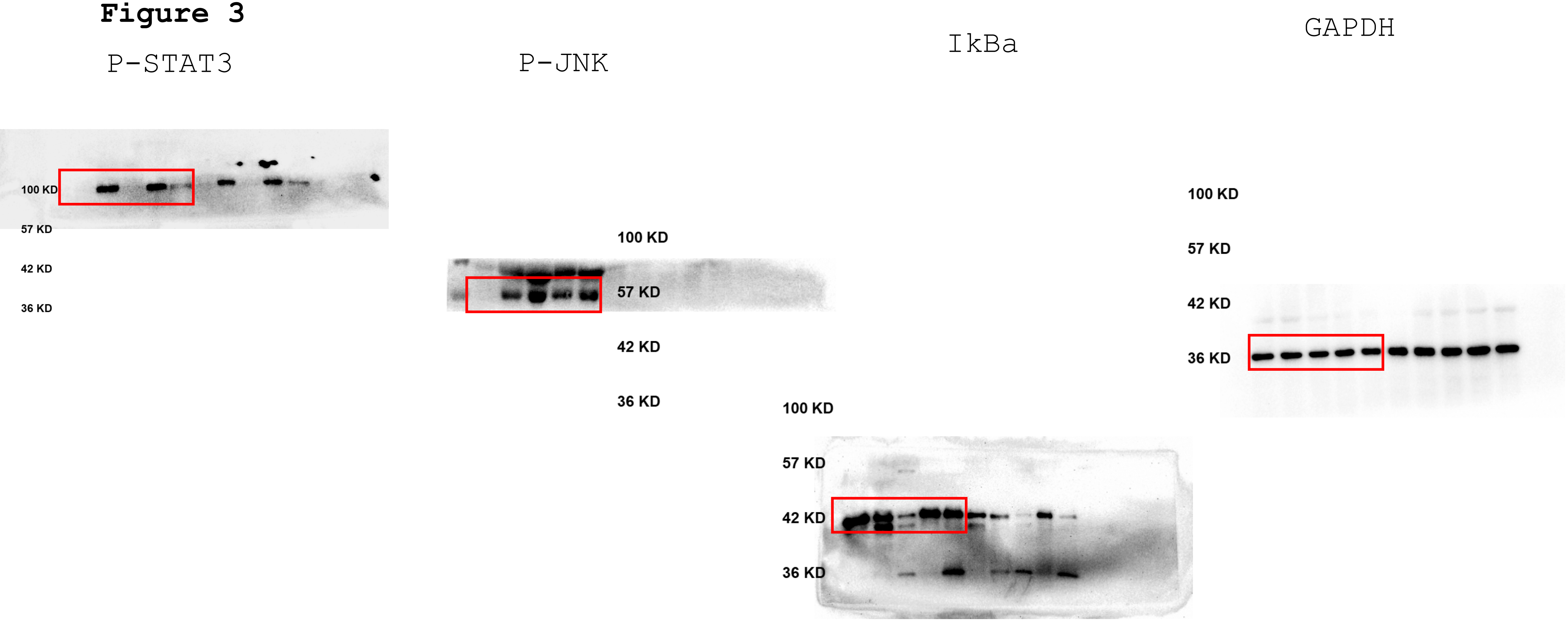

Uncorrupted WB images

Figure 4

P-STAT3

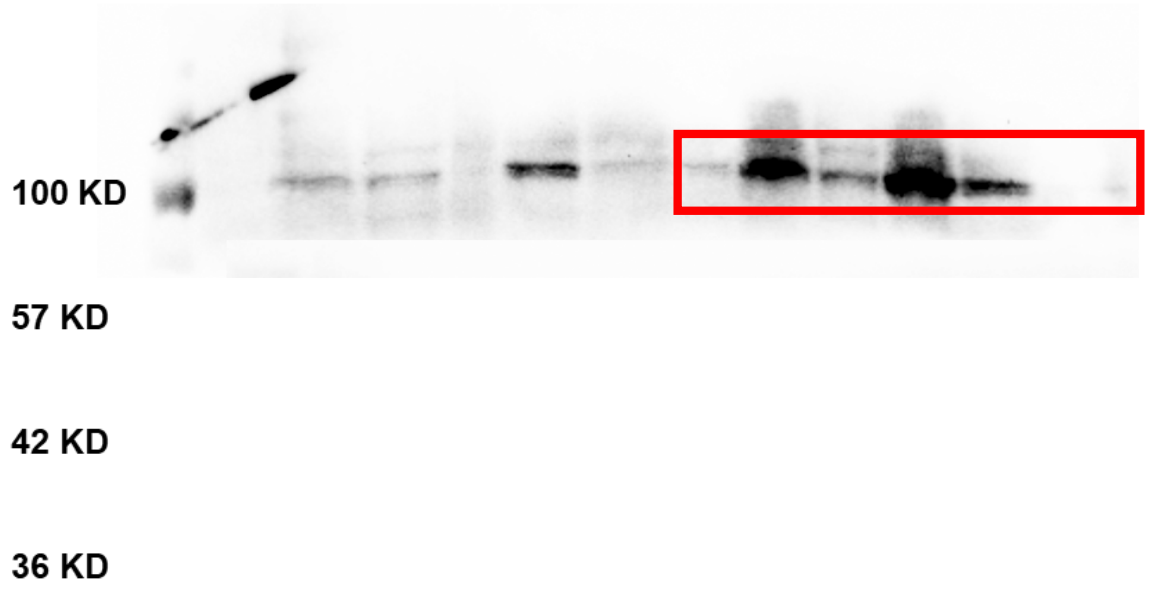

P-JNK

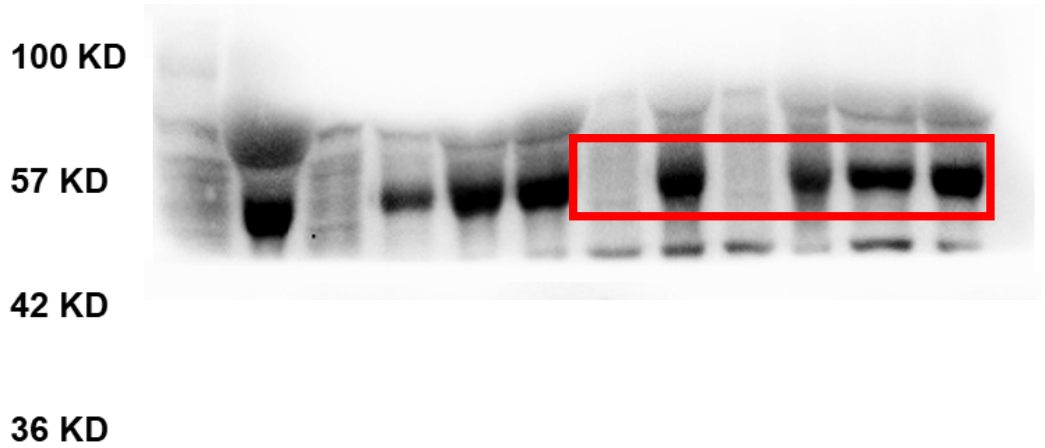

IkBa

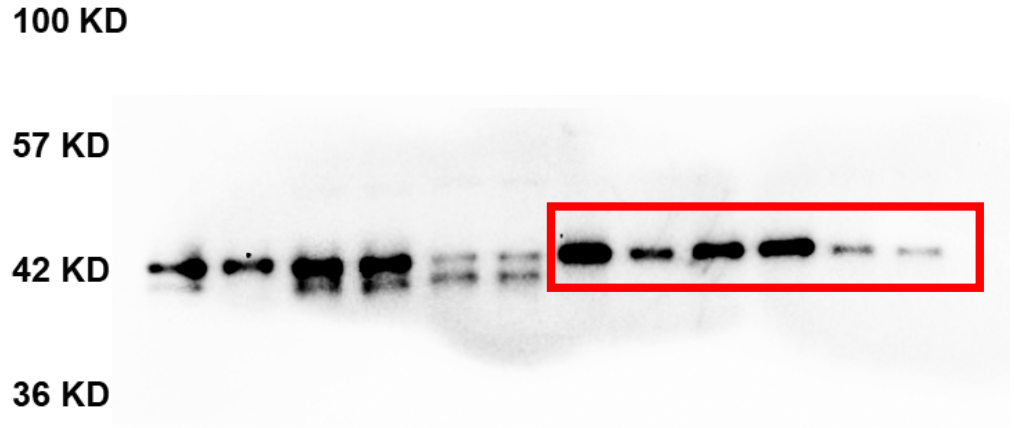

GAPDH

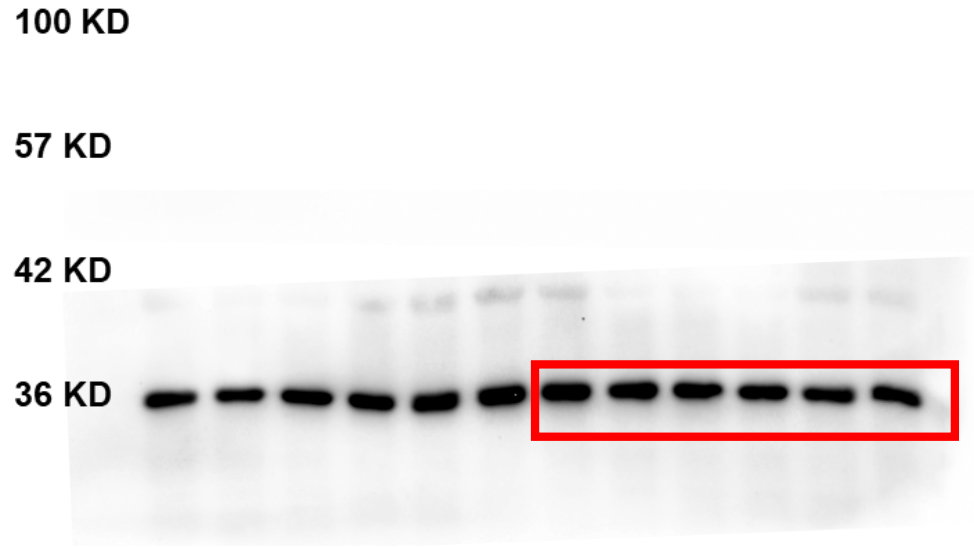

Supplement: Supplementary file 2 [file 41419_2026_9194_MOESM2_ESM.pdf]
